# Rise and Fall of Anderson Localization by Lattice Vibrations: A Time-Dependent Machine Learning Approach

**DOI:** 10.3390/e26070552

**Published:** 2024-06-28

**Authors:** Yoel Zimmermann, Joonas Keski-Rahkonen, Anton M. Graf, Eric J. Heller

**Affiliations:** 1 Department of Chemistry and Applied Biosciences, ETH Zurich, 8093 Zurich, Switzerland; 2 Department of Physics, Harvard University, Cambridge, MA 02138, USA; 3 Department of Chemistry and Chemical Biology, Harvard University, Cambridge, MA 02138, USA; 4 Harvard John A. Paulson School of Engineering and Applied Sciences, Harvard University, Cambridge, MA 02138, USA

**Keywords:** lattice vibrations, coherent states, dynamical disorder, Anderson localization, transient localization, machine learning

## Abstract

The intricate relationship between electrons and the crystal lattice is a linchpin in condensed matter, traditionally described by the Fröhlich model encompassing the lowest-order lattice-electron coupling. Recently developed quantum acoustics, emphasizing the wave nature of lattice vibrations, has enabled the exploration of previously uncharted territories of electron–lattice interaction not accessible with conventional tools such as perturbation theory. In this context, our agenda here is two-fold. First, we showcase the application of machine learning methods to categorize various interaction regimes within the subtle interplay of electrons and the dynamical lattice landscape. Second, we shed light on a nebulous region of electron dynamics identified by the machine learning approach and then attribute it to transient localization, where strong lattice vibrations result in a momentary Anderson prison for electronic wavepackets, which are later released by the evolution of the lattice. Overall, our research illuminates the spectrum of dynamics within the Fröhlich model, such as transient localization, which has been suggested as a pivotal factor contributing to the mysteries surrounding strange metals. Furthermore, this paves the way for utilizing time-dependent perspectives in machine learning techniques for designing materials with tailored electron–lattice properties.

## 1. Introduction

Anderson localization refers to the cessation of diffusive wave propagation in disordered systems [1]. On the historical front, Thouless theoretically posited [2] that at low temperatures, where inelastic processes are minimal, localization would result in higher resistance compared to that expected from ordinary elastic scattering. This insight later spurred the development of the scaling theory of Anderson localization for non-interacting electrons [3]. On the other hand, the conditions facilitating Anderson localization within an interacting system have been found to rely on several factors, including the strength of disorder, the dimensionality of the system [4], the range and type of interactions [5,6,7], and the time scales of the disorder potential dynamics [8,9].

The conundrum of whether systems localize or not was recognized early on by researchers like Gogolin [10,11], Thouless [2], and also Anderson [1,12]. For instance, the complex interplay between Anderson localization and lattice vibrations is observed in various random metal alloys and other disordered systems, such as crystalline organic semiconductors [13,14] and halide perovskites [15]. The random fluctuations caused by lattice motion gradually disrupt the quantum interference necessary for electronic state localization, leading to what has been coined transient localization (for capturing the essential aspects, see, e.g., Ref. [9]). This phenomenon combines aspects of both Anderson localized and itinerant electron systems: Electronic transport is characterized by the successive cycles of localization and delocalization à la Anderson stemming from lattice vibrations that eventually result in reduced diffusion.

Whereas Anderson localization is typically explored within the framework of a tight-binding scheme featuring random on-site energies, the standard model for lattice vibrations is established by Fröhlich, which features linear coupling between an electron and the lattice. Conventionally, lattice vibrations are viewed through a number state perspective, but the coherent state representation introduced in Ref. [16], known as quantum acoustics, treats lattice vibrations as waves rather than individual phonons. This picture utilizing the coherent state basis is a valid way to treat the lattice vibrations fully quantum-mechanically, of equal, unassailable stature to the conventional Fock (number) state approach. A quantum lattice field in a number state has a well-defined amplitude, i.e., the number of quanta, but lacks knowledge of phase. On the other hand, the field defined by a coherent state has an equal amount of uncertainty in both amplitude and phase (a more detailed discussion on the coherent states can be found, e.g., in Refs. [17,18,19,20]). However, even though these two pictures are equivalent at the most fundamental level, this duality is normally hidden by the approximations the two limits encourage. For example, a virtue of coherent states is that they are the closest quantum mechanical states to a classical description allowed by the uncertainty principle.

The quantum-acoustical perspective unveils a duality between particle and wave pictures akin to quantum optics [17,18,19,20]) established by Glauber [21]. Moreover, it allows for the electron–lattice interactions to be described in terms of a quasi-classical internal field, reminiscent of Bardeen and Shockley’s concept regarding dynamical lattice distortions in nonpolar semiconductors [22,23]. In particular, the deformation potential arising from lattice vibrations enables a quantum-coherent, nonperturbative treatment of charge carriers in coordinate space. In addition to recovering the results of the conventional Bloch-Grüneisen thory [16], the program of quantum acoustics has illuminated mysteries surrounding strange metals where transient localization plays a central role, such as T-linear resistivity at the Planckian limit surpassing the Mott–Ioffe–Regel threshold [24] and a shift in the Drude peak in the optical conductivity towards the infrared range [25]. Motivated by these advancements, we aim to identify various classes of dynamics hidden within the venerable Fröhlich model, which we express in the coherent state representation.

The quantum acoustical approach above enables the generation of large amounts of time-dependent charge carrier wavefunctions as a function of the system parameters. Clustering, a common unsupervised learning technique, provides an effective means to explore the spectrum of carrier behavior by grouping similar dynamical profiles into clusters. In general, unsupervised machine learning (ML) methods have been established as a powerful tool to identify complex patterns in large unstructured data sets [26,27,28].

In the broader landscape of ML applications in physics, our approach aligns with the recent uses of machine learning to understand and categorize complex physical phenomena, such as many-body localization and phase transitions [28,29,30,31,32,33]. However, it is important to distinguish our work from the common narrative of “using ML to do physics”. Instead, our method uses ML as a tool that complements traditional analytical and numerical methods. This distinction underscores a shift from merely applying ML techniques to physics problems towards a more integrated approach where ML assists in how we conceptualize and explore physical systems.

To the authors’ knowledge, this study is the first to apply ML techniques for analyzing the dynamics of condensed matter systems through a time-dependent lens. Moreover, our approach not only goes beyond the established focus on eigenstates but also extends the application of ML to condensed matter systems outside of tight-binding models, such as spin chains.

Our program is as follows. In Section 2, we delineate the theoretical framework across three stages. We first put forward the concept of deformation potential (Section 2.1), highlighting its significance as a palpable nonperturbative internal field for electrons (Section 2.2). To facilitate the analysis of electron–lattice dynamics, we introduce a machine learning methodology in Section 2.3. In Section 3, we present our classification of wavepacket dynamics leveraging the ML approach, exploring variations in the strength of the electron–lattice interaction and illustrating a resulting “phase diagram”. Additionally, we conduct a detailed examination of one of the identified sectors connected to transient localization. Finally, we conclude our findings and discussions in Section 4.

## 2. Theory and Methods

More explicitly, we investigate the diversity of physics contained by the following Hamiltonian:

(1) $$H_{F}=\sum_{p}\varepsilon_{p}c_{p}c_{p}^{†}+\sum_{q}\hbar \omega_{q}a_{q}^{†}a_{q}+\sum_{pq}g_{q}c_{p+q}^{†}c_{p}\left(a_{q}+a_{-q}^{†}\right)$$

where $c_{p}$$\left(c_{p}^{†}\right)$ is the creation (annihilation) operator for electrons with momentum p and energy $\varepsilon_{p}$ whereas $a_{q}$$\left(a_{q}^{†}\right)$ is the creation (annihilation) operator for longitudinal acoustic phonons of wave vector q and energy $\hbar \omega_{q}$, respectively. The electron–phonon interaction is defined by its Fourier components $g_{q}$. This Hamiltonian embodies the lattice q, the electrons p, and their lowest-order (linear) interaction that we next cast into the multimode coherent state basis of lattice degrees of freedom |χ〉.

### 2.1. Deformation Potential

The coherent state picture developed in Ref. [16] is the dual partner of the traditional number state description of electron–lattice dynamics. In this framework, each normal mode of lattice vibration with a wave vector q is associated with a coherent state |q〉. At thermal equilibrium, each mode can be considered to be equilibrated with a heat bath at temperature *T*, giving thermal ensembles of coherent states where the average occupation of the mode ${\langle n_{q}\rangle }_{\mathrm{th}}$ is given by the Bose–Einstein distribution. Employing the independence of normal modes, entire lattice vibrations can be described as the product state of the coherent states of the normal modes, in other words, as a multimode coherent state $|\chi \rangle ={⨂}_{q}|q\rangle$, as studied in Ref. [34].

Even though the Fock state perspective focusing on the particle characteristics of lattice vibrations and the coherent state viewpoint emphasizing the wave nature are formally equivalent, the approximations they inspire are vastly different. For instance, a common approach is to assume a direct product state |p〉⊗|χ〉, combining the electronic state |p〉 and the lattice state |χ〉 while neglecting entanglement effects; this approach is equivalent to employing the time-dependent Hartree approximation. Moreover, we only consider the longitudinal acoustic branch of lattice vibrations. Then, as detailed in Ref. [16], the quasi-classical limit of quantum acoustics unveils a real-space, time-dependent description of electron–lattice interaction in terms of the *deformation potential*.

(2) $$\begin{array}{c} V_{D}\left(r,t\right)=\langle \chi |\sum_{q}g_{q}\left(a_{q}+a_{-q}^{†}\right)|\chi \rangle =\sum_{\begin{array}{c} q \end{array}}^{\left|q\right|\leq q_{D}}2g_{q}\sqrt{{\langle n_{q}\rangle }_{\mathrm{th}}}\cos\left(q\cdot r-\omega_{q}t+\varphi_{q}\right), \end{array}$$

where $\varphi_{q}$ is the phase of the coherent state |q〉. Furthermore, we assume the phases $\varphi_{q}$ to be uniformly distributed random variables and employ the Debye model, assuming the linear dispersion $\omega_{q}=v_{s}\left|q\right|$, where $v_{s}$ is the speed of sound. Therefore, the time dependence of the deformation potential is governed by the following wave equation:

(3) $$\frac{\partial^{2}}{\partial t^{2}}V_{D}\left(r,t\right)=v_{s}^{2}\nabla^{2}V_{D}\left(r,t\right).$$

The acoustic lattice disorder field above appears as a chaotic sea of roaming sound waves, which can be loosely viewed as a dynamic, multi-wavelength adaptation of the Berry potential examined in Ref. [35], named for its association with the random wave conjecture [36] in the field of quantum chaos. On the other hand, the deformation potential stemming from lattice vibrations has a close resemblance to the vector potential of a blackbody field as first identified by Hanbury Brown and Twiss [37], except for the existence of the ultraviolet cutoff given by the Debye wavevector $q_{D}$ originating from the minimal lattice spacing *a*.

The deformation potential in itself is a peculiar object. For instance, it is homogeneously random, meaning that the probability distribution of potential values $V_{D}$ does not depend on a position r or time *t* (with the assumption of random phases). Therefore, each spatio-temporal patch of the potential is statistically indistinguishable from another. The typical length scale of the spatial correlations is determined by its largest wavenumber components $∼q_{D}$. Similarly, the typical timescale of the potential change is determined by its largest frequency components $∼\omega_{D}$. This special type of spatial-temporal correlation sets the deformation potential apart from other types of lattice distortions, as commonly investigated in the context of Anderson localization [1].

**Figure 1:**
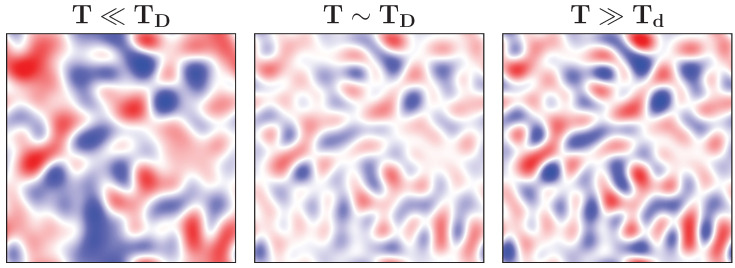
Snapshots of the deformation potential at three different example temperatures. The left and middle panels demonstrate the awakening of new vibrational modes with increasing temperature, giving rise to finer details in the potential. At the same time, the bumps (red) and dips (blue) of the potential become higher and deeper. On the other hand, when the Debye temperature is reached, all the modes are active, and the potential features grow as ∼$\sqrt{T}$, as shown in the right panel. For the sake of illustration, the left panel has a different color scale than the middle and right panels.

Even though the deformation potential overall averages to zero, its root mean square characterizing the strength of lattice disorder fluctuations grows in temperature as

$$V_{\mathrm{rms}}^{2}=\frac{2E_{d}^{2}\hbar }{\pi \rho v_{s}}\int_{0}^{q_{D}}\frac{q^{2}dq}{e^{\hbar v_{s}q/k_{B}T}-1}∼\left\{\begin{array}{c} {\left(k_{B}T\right)}^{1/2},\mathrm{when}T\gg T_{D}\\ {\left(k_{B}T\right)}^{3/2},\mathrm{when}T\ll T_{D} \end{array}\right.,$$

where $E_{d}$ is the deformation potential constant related to the coupling $g_{q}$, and ρ is the mass density of the underlying crystal lattice. As the temperature nears the Debye temperature $T_{d}$, previously dormant vibrational modes start to awaken from their Bose–Einstein slumber. This activation not only enhances the peaks and valleys as ∼$T^{3/2}$ but also brings forth finer wavelength details in the deformation potential, as depicted in the left and middle panels of Figure 1. At a temperature $T∼T_{D}$, all the possible lattice modes are in play, after which no new wave characteristics emerge. The existing potential bumps and dips simply become more pronounced as ∼$\sqrt{T}$, as illustrated via the middle and right panels of Figure 1.

### 2.2. Electron Dynamics

The time-varying deformation potential virtually demands quantum wavepacket propagation techniques for the electron. Here, we focus on the time-dependent Hamiltonian

$$\begin{array}{c} H_{0}=\frac{{\left|p\right|}^{2}}{2m^{*}}+V_{D}\left(r,t\right), \end{array}$$

where $m^{*}$ is the effective (band) mass of the electron and $V_{D}\left(r,t\right)$ is the deformation potential given by Equation (2). This effective Hamiltonian $H_{0}$ represents the electron component of the Fröhlich model defined previously in Equation (1) within the framework of the effective mass approximation.

Our investigation of electron dynamics under the defined effective Hamilton $H_{0}$ approaches the issue from the point of view of Gaussian wavepackets that are a common tool for analyzing time-dependent aspects of a quantum system [38,39], for instance in the studies of quantum optics [17,18], scarring [40,41,42], and branched flow [43,44,45]. Here, we choose the following test Gaussian for representing the charge carrier:

(4) $$\Psi \left(r,0\right)=N\exp\left(\frac{1}{4}{\left|r\cdot \sigma \right|}^{2}-ik\cdot r\right),$$

where N is the normalization factor, and $\sigma =(\sigma_{x}^{-1},\sigma_{y}^{-1})$ describes the initial width of the wavepacket. Without loss of generality, we can choose to launch the test wavepacket into the x direction with the Fermi momentum, thus $k=(k_{F},0)$, where $k_{F}$ is the Fermi wavevector. The memory of the initial form of the wavepacket is quickly lost in the chaotic potential and its exact form is unimportant.

To propagate the wavepacket in time, we utilize the third-order split operator method [38,39,46,47] applied to the time-dependent Schrödinger equation:

(5) $$\begin{array}{c} i\hbar \frac{\partial }{\partial t}\Psi \left(r,t\right)=H_{0}\Psi \left(r,t\right). \end{array}$$

**Figure 2:**
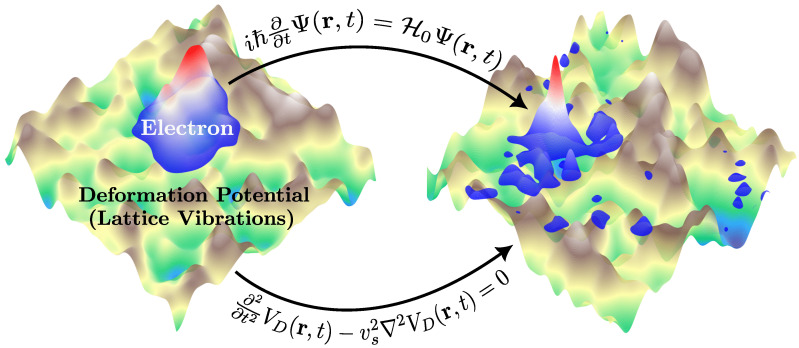
The quantum acoustical Wave-on-Wave (WoW) approach to charge carrier dynamics. An electron wavepacket propagates atop a deformation potential, which itself evolves according to the wave equation. As it traverses this shifting acoustic landscape shaped by acoustic deformations, the electron undergoes quasi-elastic scattering akin to impurity scattering.

Figure 2 illustrates the charge carrier wave, originally a Gaussian as described in Equation (4), evolving under the influence of the dynamic lattice wave field, which converts the always-accessible wave nature of lattice vibrations into something valuable, a point where the quantum-acoustical perspective becomes tangible. Within this kind of Wave-on-Wave (WoW) approach, as detailed in Refs. [24,25], one winds up solving two interacting equations of motions simultaneously: one for the lattice and one for the electron (i.e., the time-dependent Schrödinger equation for the charge carrier and the wave equation for the lattice vibrations).

In general, we can crudely characterize the WoW dynamics based on two criteria:

$$\bar{K}=\frac{\hbar^{2}k_{F}^{2}}{2m^{*}V_{\mathrm{rms}}}\left\{\begin{array}{cc} \gg 1 & \to \mathrm{Perturbative}\\ ≲1 & \to \mathrm{Nonperturbative}, \end{array}\right.\;\mathrm{and}\bar{\lambda }=\frac{\pi }{k_{F}a}\left\{\begin{array}{cc} \ll 1 & \to \mathrm{Incoherent}\\ ≲1 & \to \mathrm{Coherent}. \end{array}\right.$$

Here, we want to point out that the term “coherence” is reserved to describe the spatial phase coherence of the electron wavefunction, not to be conflated with the “coherent versus incoherent metals” nomenclature, which pertains to the breakdown of the quasi-particle paradigm. Rather, this criterion refers to the quantum coherence of electrons that becomes important in scattering when the wavelength of the electrons (Fermi wavelength) is not much less than twice the lattice constant *a*. The WoW approach presented here adeptly captures the persistence of coherence between successive collisions, a facet commonly overlooked by conventional Boltzmann transport methods. Indeed, the preservation of coherence beyond the first scattering event can wield significant influence, as evidenced by Refs. [16,24].

On the other hand, the comparison of the kinetic energy of the electron (a fair approximation is given by the Fermi energy) with the root mean square of the deformation potential determines whether the lattice vibration and its resultant electron scattering can be treated perturbatively or not. In essence, the deformation potential cannot be merely considered a minor perturbation to the free-electron model Hamiltonian. Instead, it can result in a substantial effect on the electronic density of states, as shown in Refs. [16,48].

### 2.3. Clustering

In addition to this classification based on the static properties of the electron–lattice interaction, we here explore the dynamical aspects of this relationship by examining two distinct measures, namely the mean squared displacement (MSD) and inverse participation ratio (IPR). The spread of the wavepacket over time is measured by the MSD:

(6) $$\begin{array}{c} \alpha \left(t\right)=\int \Psi^{*}\left(r,t\right){\left[\langle r\rangle -r\right]}^{2}\Psi \left(r,t\right)dr. \end{array}$$

Moreover, we assess the level of wavepacket localization by considering the IPR

(7) $$\begin{array}{c} \beta \left(t\right)={\int |\Psi \left(r,t\right)|}^{4}dr, \end{array}$$

a widely used method for analyzing scarred states or Anderson localized states in a disordered medium [49]. Here, it is important to note that the measures discussed here deviate from their conventional definition by being determined as a function of time rather than time-independent, as typically seen in the studies of eigenfunctions. Furthermore, we can combine the time-evolving quantities into one two-dimensional times series, denoted as

$$\begin{array}{c} F\left(t\right)=\left(\begin{array}{c} \alpha (t)\\ \beta (t) \end{array}\right), \end{array}$$

which paves the way to leveraging ML methods for time series to discern various transport regimes.

Specifically, we apply *k*-means [50] clustering using dynamic time warping [51] to the mean-variance normalized set of series {F(t)}, which consist of 50 timesteps with each timestep representing 2fs of evolution, across different system variable settings. At its core, *k*-means is an algorithm to solve the optimization problem of partitioning a given set into *k* clusters such that the in-cluster variance is minimized (for a detailed explanation of *k*-means and our method, we refer the reader to Appendix A). It is important to stress that this optimization is performed in a fully unsupervised manner, meaning that it only processes the raw time series {F(t)} and is blind to the system variables used to generate the data. To ensure robustness and to mitigate the effect of statistical fluctuations, we average the clustering results of an ensemble of 10 time series data sets, each generated using randomly initialized deformation potentials. This method allows us to objectively identify unique clusters corresponding to diverse dynamical regimes hidden within the Fröhlich Hamiltonian.

## 3. Results

As a real-world example of electron–lattice dynamics, at least within the Fröhlich Hamiltonian, we investigate the prototypical strange metal lanthanum strontium copper oxide (LSCO), renowned for its diverse physics [52]. This canonical cuprate, discovered by Bednorz and Muller [53], is characterized by an orthorhombic space group [54]. The main electrical transport occurs between the ${\mathrm{CuO}}_{2}$ layers, making it effectively two-dimensional in nature [52]. Furthermore, LSCO has a large electron–lattice coupling, and its Fermi energy is adjustable through doping. The material parameters for optimal doping are given in Table 1 based on experimental values derived from Refs. [55,56,57,58,59]. These serve as the basis for constructing an associated deformation potential. These parameters align with previous investigations on strange metals [24,25] using a quantum-acoustical perspective. Here, we explore electron–lattice dynamics in a broader scope, rather than focusing on specific attributes like electrical conductivity.

To enable this analysis, we introduce two scaled variables that we vary in our simulations: dimensionless temperature $\tilde{T}=T/T_{D}$ and effective electron–lattice coupling $\tilde{G}=2k_{F}/q_{D}$. The coupling is adjusted by varying the Fermi wavevector $k_{F}$ (energy) of the electron while maintaining the underlying lattice structure constant (with a fixed Debye wavevector $q_{D}$). This ensures that our variables $\tilde{T}$ and $\tilde{G}$ are independent, a premise supported by the evidence from our simulations.

### 3.1. Phase Diagram

**Figure 3:**
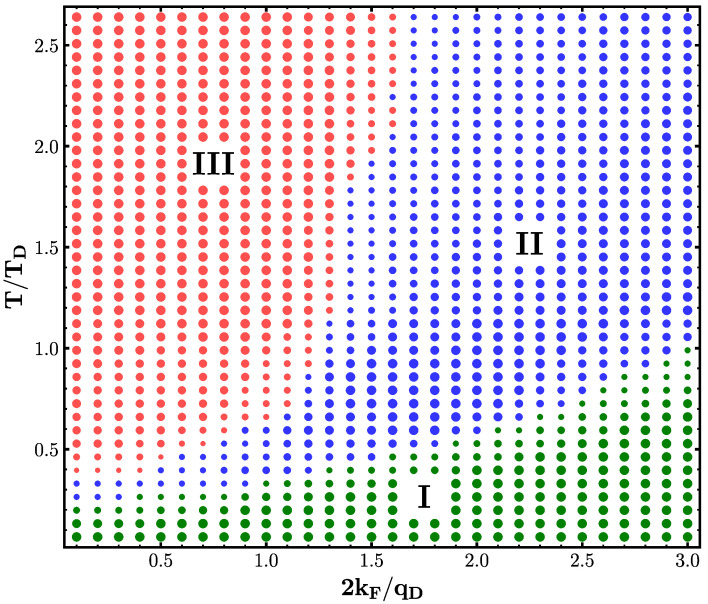
Phase diagram of LSCO in the dynamic potential field. The phase diagram was derived using a machine learning-based clustering algorithm to analyze time series data of the wavefunction evolution within systematically varied deformation potentials. This analysis involved variations in temperature $\tilde{T}$ and effective coupling $\tilde{G}$; every point corresponds to one unique configuration. The following three clusters were identified: (I) refractive scattering region (green), (II) diffraction behavior (blue), and (III) short-time localization at high temperatures (red). The size of the points indicates the level of agreement across an ensemble of different wavefunction data sets.

Our central finding is presented in Figure 3 showing the dynamical data classified using the ML-based clustering algorithm explained above and as detailed in Appendix A as the temperature $\tilde{T}$ and coupling strength $\tilde{G}$ are varied. Three distinct phases are identified as labeled by the differently colored regions. We want to emphasize that the term “phase” is used here to refer to regimes of different dynamical behavior, not in the thermodynamical sense. There are no sharp boundaries between these phases; the changes are gradual rather than true phase transitions. This fact is highlighted in Figure 3 by the different sizes of the points, representing the level of agreement within the ensemble of studied wavepackets for the given parameters.

**Figure 4:**
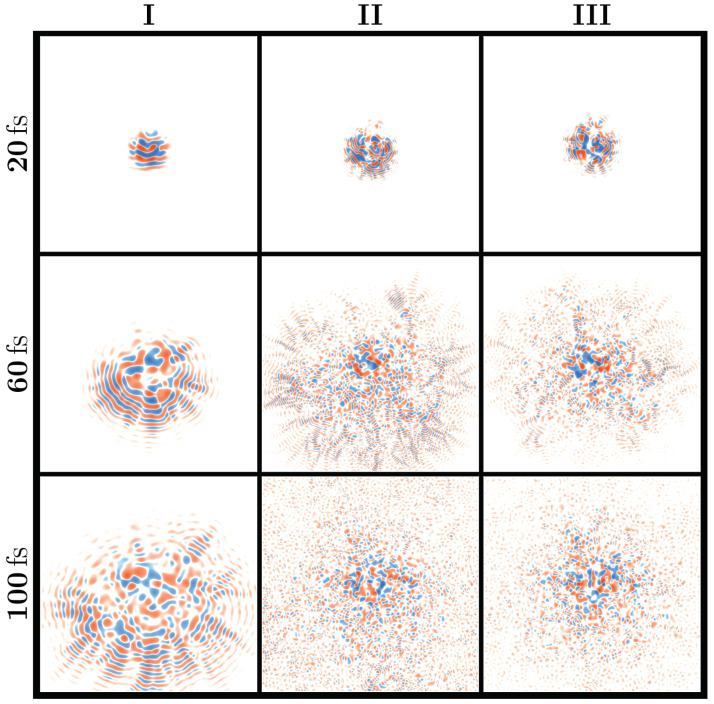
Evolution of electronic wavefunctions in dynamic deformation potentials. This chart displays the real part of the wavefunction in two-dimensional coordinate space, where red and blue colors indicate positive and negative amplitudes, respectively. Each column represents parameters selected as examples from the identified clusters I, II, and III. For each cluster, the panels arranged vertically from top to bottom show snapshots of the wavefunction at increasing times of 20 fs, 60 fs, and 100 fs.

We interpret the distinct regions as follows: refractive scattering phase (I), diffraction behavior phase (II), and transient localization phase (III). We present three zones of characteristic wavepacket evolution, selected to represent the dynamical behavior of each phase. The snapshots in Figure 4 depicts the real part of the evolution of a common initial Gaussian wavepacket at times of 20fs, 60fs and 100fs under three different conditions of temperature $\tilde{T}$ and coupling strength $\tilde{G}$.

Phase I (green) is characterized by an almost linear phase boundary starting at $\tilde{T}\approx 0.45$ rising across the range of $\tilde{G}$ explored. This phase is perturbative in the sense of $\tilde{K}\gg 1$. As seen in the left column of Figure 4, the scattering of the wavepacket is mainly refractive. This trend will lead to branched flow behavior [43] at longer times; a propagating wave forms tree-like branches under a weakly disordered medium, due to small-angle refraction [60]. Moreover, there is a partial transparency of the electrons to any shorter wavelength modes ($q>2k_{F}$) present in the underlying deformation potential, as is further explained in Ref. [16].

Phase II (blue) covers the upper right section of the phase diagram with high values of temperature variability ($\tilde{T}$) and effective coupling($\tilde{G}$) and is separated by an exponential-like phase boundary from Phase III (red), which is characterized by high temperatures but lower $\tilde{G}$ levels. Like Regime I, the second phase is characterized by relatively adiabatic lattice dynamics. In other words, the deformation landscape appears as if it is stationary for an electron, at least for short times of ∼$2\pi /\omega_{D}$. This fact is further confirmed by our IPR results below. Furthermore, this regime is perturbative but also classical-like, meaning that the wavelength of the electron is shorter than the effective shortest length scale of the deformation potential. As thoroughly discussed in Ref. [16], the perturbation theory pathway, particularly Fermi’s golden rule, is proven to be highly successful in this phase.

The final phase (Phase III) identified by the ML-clustering is associated with highly nonperturbative ($\tilde{K}≲1$) electron–lattice interaction, primarily existing in the parameter space where electron dynamics can be considered as coherent ($\tilde{\lambda }≲1$). Therefore, wave interference and diffraction effects are important because the electron wavelength is larger than the shortest length scale of the deformation potential. Notably, this phase begins at low temperature as $\tilde{T}\approx 0.5$ while extending to very high temperatures across the range of $\tilde{T}$ investigated.

In Phase III, as depicted in the right column of Figure 4, an initial wavepacket encounters significant scattering from a strong deformation potential, initially causing diffusive behavior akin to that seen in Phase II. However, wavepacket spreading eventually ceases due to quantum interference effects, signifying an onset of localization. Nevertheless, the random fluctuations introduced by the motion of the lattice slowly but surely scramble the quantum interference required for the long-term confinement of the wavepacket, resulting in the transient nature of this localization (for capturing the essential aspects of this phenomenon, see, e.g., Ref. [9]).

To achieve a more comprehensive understanding, we adopt a static potential approximation, wherein the temporal aspect of the lattice deformation field of Equation (2) is neglected, effectively frozen into its original configuration. Within this frozen potential framework, we carry out an analysis analogous to that of the evolving deformation potential as above. This is outlined in Appendix B. Employing the same cluster classification, we categorize the data of an electron wavepacket evolution under a static deformation potential, yielding a phase diagram similar to its dynamic counterpart in Figure 3, albeit with slightly sharper phase boundaries. This comparison validates treating the deformation potential as predominantly static, particularly in Phases I and II. On the contrary, in Phase III, the static deformation potential results in full Anderson localization of the wavepacket that proves transient when the deformation potential undergoes morphing and undulation over time, as further elucidated in the subsequent analysis.

### 3.2. Transient Localization

In this section, we delve deeper into the nature of transient localization induced by lattice vibrations taking place within Phase III. At timescales shorter than the characteristic timescale of $2\pi /\omega_{D}∼100\mathrm{fs}$, lattice vibrations mimic a static, internal disorder field, triggering the onset of Anderson localization. Therefore, in reference to the dynamical field where the motion of the lattice disrupts the process of Anderson localization, we are also exploring the localization behavior of a wavepacket within a frozen potential approximation. In both cases, we quantify the level of localization by studying the time-dependent IPR, denoted as α(t) in Equation (7), and introducing a subsidiary product of the MSD, called the instantaneous diffusivity

(8) $$\begin{array}{c} D\left(t\right)=\frac{1}{4}\frac{d\beta (t)}{dt}, \end{array}$$

where β(t) is defined in Equation (6).

**Figure 5:**
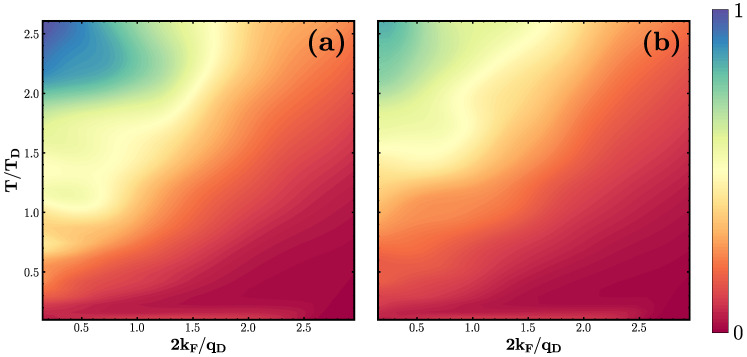
Inverse scattering rate 1/τ for electrons in LSCO in a (**a**) frozen and (**b**) dynamic deformation potential on a normalized scale. Both scenarios exhibit a high inverse scattering rate region in the upper left corner (Phase III), suggesting strong constraints on carrier mobility. This region is markedly more prominent in the frozen potential scenario, as the lattice vibrations in the dynamic potential continuously disrupt short-lived attempts at localization. The shape of counter lines closely resembles the dynamical phase transition lines depicted in Figure 4.

We begin by examining the instantaneous diffusivity, which then determines the diffusion constant *D* as its long-term value, i.e., $D=\lim_{t\to \infty }D\left(t\right)$. In the spirit of the Einstein and Drude models, we can convert this diffusion constant into an inverse scattering rate as

$$\frac{1}{\tau }=\frac{k_{B}T}{m^{*}D},$$

consistent with the definition used in Ref. [24]. Figure 5 shows the inverse scattering rate 1/τ for both the cases of frozen (left panel (right panel) deformation potential. Overall, the analysis of the scattering rate supports the ML classification underlying the phase diagram shown in Figure 4. Both scenarios exhibit a notably high inverse scattering rate within Phase III of the phase diagram (upper right corner), indicating significant constraints on carrier mobility, as expected in the context of Anderson localization. This effect is more prominent in the frozen potential approximation than in the case of the morphing deformation potential, underlining the fact that the dynamics of the deformation potential continuously disrupt short-lived localization attempts. Moreover, we observe that the contour lines of the inverse scattering time in Figure 5 closely resemble the phase boundaries seen in Figure 4.

**Figure 6:**
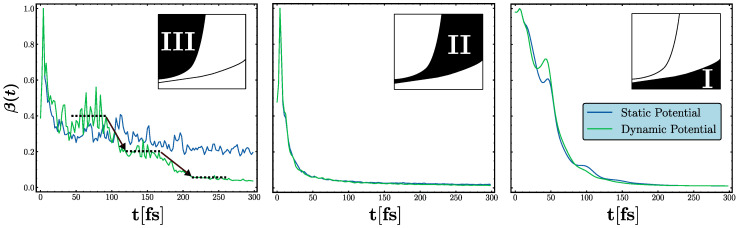
Normalized inverse participation ratio β(t) of the wavepacket as a function of time across three distinct dynamical regions identified in the machine learning-based phase diagram shown in Figure 4. Figure contrasts the behaviors under static (blue line) and dynamic (green line) deformation potentials. In Phase III (left), brief localization periods (indicated by dashed lines) are broken by lattice motion (indicated by arrows), while full localization, indicated by the saturation of β(t), occurs within the frozen potential approximation. In Phase II (middle) and Phase I (right), the decay of β(t) follows a relatively exponential trend without signs of localization as observed in Phase III. Notably, the decay is faster in Phase I when compared to Phase II, where small oscillations linked to quantum interference are present.

A deeper insight into the emergence of transient localization is obtained by investigating the IPR of the wavepacket evolving over time. In the left panel of Figure 6, we show the evolution of the IPR in Phase III for both static (blue) and dynamic (green) deformation potentials. In the approximation where the deformation potential remains static at its initial state, the IPR stabilizes at a certain value (β∼0.3) after an initial decrease, heralding the Anderson localization of the wavepacket. Similarly, when the wavepacket is subject to a dynamic deformation potential, a form of Anderson localization occurs. This localized state is eventually disrupted by lattice motion, leading to a temporary delocalization followed by a brief relocalization before being disintegrated again by potential evolution. This cyclical process of plateauing IPR seen in the left panel of Figure 6 epitomizes the birth and demise of the Anderson localization due to lattice vibrations.

We would like to point out that the dynamics of short wavelength components of the deformation potential can significantly influence localization within a time window shorter than the characteristic time $\omega_{D}/2\pi$. For example, the left panel of Figure 6 demonstrates an instance of dynamically enhanced localization: the random initial configuration of the potential creates valleys (mountains) that quickly move towards (away) the wavefunction, causing boosted localization. Vice versa, these types of small potential variations in time can also lead to weaker localization compared to the static potential case in the first localization plateau seen in the left panel of Figure 6.

To provide a full picture, we also present the evolution of the IPR measure for Phase I and II in the right and left panels of Figure 6, respectively. Neither phase exhibits any signs of localization, contrasting with the behavior observed in Phase III. Moreover, the overlapping curves of the static and dynamic potentials further support the earlier assertion that the deformation potential can be effectively approximated as a static entity in Phase I and II. In Phase II, the IPR shows rapid exponential decay, quickly approaching the ergodic (fully delocalized) limit of β∼0. This behavior resembles that of a system with a high density of impurities characterized by Gaussian statistics. Similarly, in Phase I, the IPR decreases towards the ergodic limit, albeit at a slower rate, displaying subtle oscillation. The slower, non-exponential decay can be attributed to the weak, refractive nature of wavepacket scattering, in conjunction with quantum coherence and interference effects.

## 4. Conclusion and Future Directions

Quantum acoustics opens up an unexplored pathway to investigating the intricate Fröhlichian electron–lattice interaction inaccessible with the standard methods of perturbation theory. We take the concept further by treating it not just as a dual perspective on lattice vibrations, but as a versatile tool in ascendance: a time-dependent, nonperturbative approach for electron–lattice interaction in coordinate space. Moreover, in the quasi-classical limit of the coherent state formalism, the quantum-acoustical way unveils the dynamics of electrons navigating through an internal lattice disorder field undulating and propagating in time.

In particular, we have here demonstrated the efficacy of unsupervised machine learning techniques in categorizing and analyzing the intricate aspects of electron dynamics stemming from lattice vibrations. Specifically, we have unraveled three distinct phases of behavior: refractive scattering, diffraction, and transient localization. Subsequently, we have assayed the latter phase, where the Anderson localization attempts of electron wavepackets are periodically disrupted by lattice movement, further enlightening an enigmatic phenomenon suggested to underpin the mysteries surrounding strange metals.

Our study, supported by machine learning, explores the parameter space characterized by temperature and effective coupling, focusing on the paradigmatic strange metal LSCO, known for its two-dimensional transport behavior. However, the presented method can be readily extended to variations in any set of material parameters—potentially augmented by density functional simulations as they are not necessarily independent—and generalized to electron–lattice dynamics in three dimensions. Therefore, our work not only lays the groundwork for uncovering hidden realms in electron–lattice interaction but is also a testament to designing materials with customized features by employing machine learning techniques from a dynamics perspective.

## Figures and Tables

**Table 1:** Material parameters for LSCO that are used in the simulations for constructing the deformation potential.

Parameter	*n*	$m^{*}$	$v_{s}$	$E_{d}$	ρ	$E_{F}$	*a*	$T_{D}$
	$\left[10^{27}m^{-3}\right]$	$\left[\left(m_{e}\right)\right]$	[m/s]	[eV]	$\left[10^{-6}\mathrm{kg}/m^{2}\right]$	[eV]	[Å]	[K]
LSCO	7.8	9.8	6000	20	3.6	0.12	3.8	379

**Figure A1:**
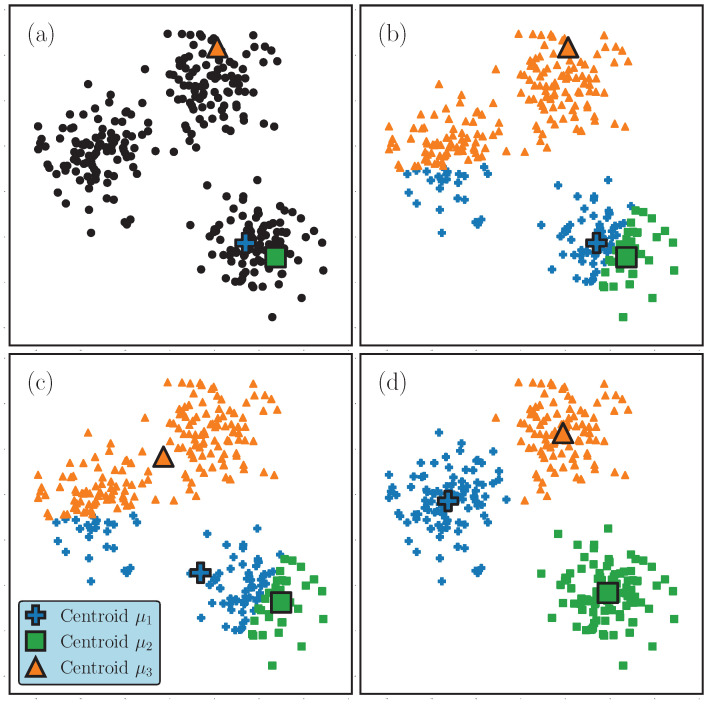
Toy example to illustrate a run of the *k*-means algorithm with hyperparameter k=3 on example data in $R^{2}$ (black dots). (**a**) Initial centroids are chosen by an initialization scheme (here: random initialization). (**b**) Assignment step of the first iteration. (**c**) Update step of the first iteration. (**d**) Final result. The algorithm converges after two consequent iterations yield sufficiently similar clusters (here the 10th iteration).

**Figure A2:**
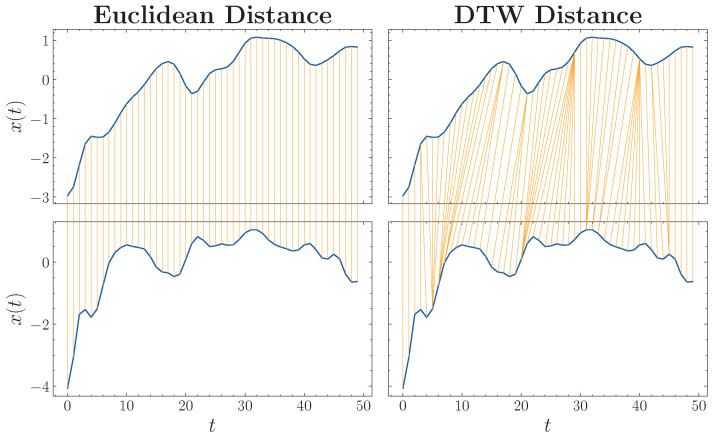
Illustration of the difference between the Euclidean distance and Dynamic Time Warping (DTW) distance for two example MSD sequences from our data set. While the Euclidean distance compares values at corresponding time steps, DTW temporally aligns the sequences, providing a more robust distance measure for time series data.

**Figure A3:**
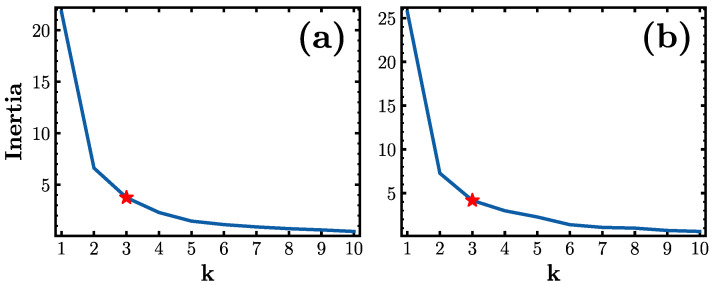
Elbow plot depicting the sum of squared distances of samples to their nearest cluster center for varying values of *k* for the (**a**) frozen and (**b**) dynamic lattice vibration field scenario. The elbow point (indicated by a red star), where the curve shows a distinct bend, suggests the optimal number of clusters for our analysis, as increasing the number of clusters beyond this value yields diminishing returns in terms of model improvement.

**Figure A4:**
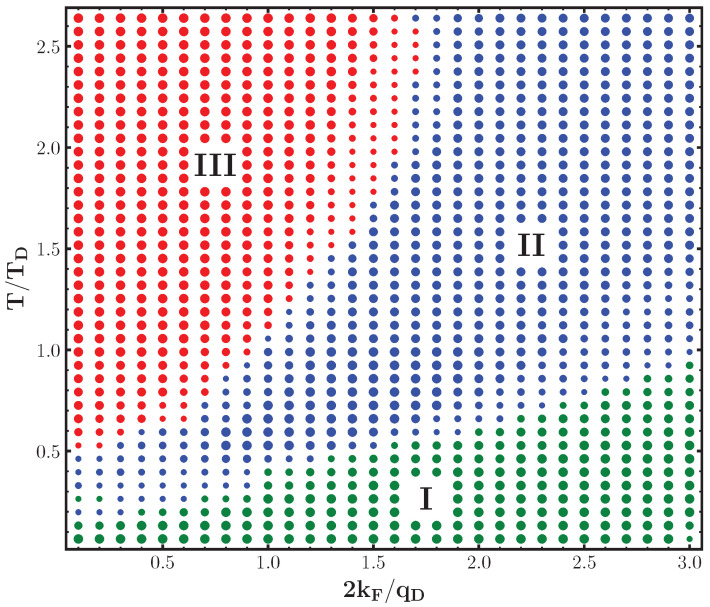
Phase diagram of LSCO in the frozen potential field. The phase diagram was derived using a machine learning-based clustering algorithm to analyze time series data of the wavefunction evolution within systematically varied deformation potentials. This analysis involved variations in temperature $\tilde{T}$ and effective coupling $\tilde{G}$; every point corresponds to one unique configuration. The following three clusters were identified: (I) refractive scattering region (green), (II) diffraction behavior (blue), and (III) Anderson localization (red). The size of the points indicates the level of agreement across an ensemble of different wavefunction data sets.

## Data Availability

The data that support the plots within this paper and other findings of this study are available from the corresponding author (Y.Z.) upon reasonable request.

## Appendix A. Clustering Analysis

For all cluster analyses in this work, the *k*-means implementation in the TimeSeriesKMeans module of the tslearn package [61] was used. We give a brief overview of the method here.

### Appendix A.1. k-Means

The goal of the *k*-means algorithm, which is attributed to Lloyd [50], is to divide $X=\{x_{1},x_{2},\ldots ,x_{N}\}$ with $x_{i}\in R^{n}$ into disjoint sets (clusters) $S=\{S_{1},S_{2},\ldots ,S_{k}\}$, $S_{i}\subseteq X$, while minimizing the in-set variance. More concretely, one aims to find

(A1) $$\begin{array}{c} \underset{S}{\arg\min}L\left(S\right)\equiv \underset{S}{\arg\min}\sum_{i=1}^{k}\sum_{x_{j}\in S_{i}}{∥x_{j}-\mu_{i}∥}^{2}=\underset{S}{\arg\min}\sum_{i=1}^{k}\left|S_{i}\right|\mathrm{Var}\left[S_{i}\right], \end{array}$$

where ∥·∥ is the Euclidean norm, $|S_{i}|$ denotes the cardinality of $S_{i}$, i.e., the size of cluster $S_{i}$, and $\mu_{i}$ is the cluster center (*centroid*) of the *i*th cluster, defined as follows:

(A2) $$\begin{array}{c} \mu_{i}=\frac{1}{|S_{i}|}\sum_{x_{j}\in S_{i}}x_{j} \end{array}$$

After some initialization scheme places the initial centroids, the algorithm alternates between two steps:

1. Assignment Step: Assign all $x_{i}$ to their closest centroid, as measured by the squared Euclidean distance. This defines the cluster memberships S.
2. Update Step: Update the centroids using S according to Equation (A2).

It is easy to see that these steps cannot increase the in-set variance; the algorithm is guaranteed to converge. However, there is an important caveat in that the objective function L(S) defined in Equation (A1) is non-convex and the algorithm can therefore converge to a local optimum that is not the global minimum. This problem can be mitigated by an intelligent choice of initialization scheme, most commonly *k*-means++ [62], and by running the algorithm multiple times and taking the clustering with the minimal L(S) post-convergence [27]. An example run of *k*-means on toy data in $R^{2}$ is shown in Figure A1.

### Appendix A.2. Dynamic Time Warping

When working with time series data $x\left(t\right)=\left(x^{t_{1}},x^{t_{2}},\ldots ,x^{t_{M}}\right)$, $x^{t_{i}}\in R^{d}$, one could naively embed x(t) in the space $R^{M\times d}$ (here, M=50 and d=2) equipped with the Euclidean metric and run *k*-means exactly as described above. However, this approach has significant limitations. It only works for sequences of equal length, and more critically, the Euclidean metric is unable to account for the temporal nature of the sequences. By operating solely on elements with the same time indices, it ignores potential temporal misalignment between sequences. Consequently, even time series with similar features can result in a large metric distance if their phases differ. This makes it an ineffective measure for determining similarity. Dynamic time warping (DTW), introduced by Sakoe and Chiba [51], has been employed to overcome these challenges by identifying the most temporally appropriate pairs of time indices for comparison [63]. Formally,

(A3) $$\begin{array}{c} \mathrm{DTW}\left(x\left(t\right),\;y\left(t\right)\right)=\min_{\pi }\sqrt{\sum_{(t_{i},t_{j})\in \pi }{∥x^{t_{i}}-y^{t_{j}}∥}^{2}} \end{array}$$

where the time index pairs $\pi =\{\pi_{0},\pi_{1},\ldots ,\pi_{K}\}$ satisfy certain (boundary) conditions that we will not elaborate on for brevity (for an extensive introduction to the topic, see, e.g., Ref. [64]). One thus ends up with a new, warped time path for the comparison of the time series, as is illustrated in Figure A2.

DTW is not a metric, because DTW(x(t),y(t))=0 does not imply x(t)=y(t) and it does not obey the triangle inequality. Nevertheless, the Fréchet mean, which generalizes Equation (A2) to any metric space, is used to compute a centroid-like representation, commonly referred to as barycenter:

(A4) $$\begin{array}{c} \mu_{i}\left(t\right)=\underset{z(t)}{\arg\min}\sum_{x_{j}\left(t\right)\in S_{i}}\mathrm{DTW}{\left(z\left(t\right),x_{j}\left(t\right)\right)}^{2} \end{array}$$

This problem is computationally NP-hard [65]. DTW Barycenter Averaging (DBA), proposed by Petitjean et al. [66], is a widely used algorithm to approximate the barycenters.

### Appendix A.3. Feature Scaling

Since *k*-means depends on distances, it is important to scale the features before performing the optimization. In this analysis, we normalize the features to zero mean and unit variance in the time dimension:

$$\begin{array}{c} \tilde{x}\left(t\right)=\frac{x\left(t\right)-\mu_{t}}{\sigma_{t}}, \end{array}$$

where $\mu_{t}=\frac{1}{T}\int_{0}^{T}x\left(t\right)dt$ and $\sigma_{t}=\sqrt{\frac{1}{T}\int_{0}^{T}{\left(x\left(t\right)-\mu_{t}\right)}^{2}dt}$.

### Appendix A.4. Choosing k

The parameter *k*, i.e., the number of clusters, has to be set before performing the optimization Equation (A1). To find a suitable *k* for our analysis, we run the *k*-means algorithm multiple times with varying values of *k* ranging from 1 to 10. Figure A3 shows a plot of the sum of squared distances of samples to their nearest cluster center (inertia) against different values of *k*. The point where the curve bends or forms an elbow is considered an indicator of the natural number of clusters in the underlying data [27]; additional clusters beyond this point do not substantially improve the model’s representation of the different dynamical regimes of the system.

### Appendix A.5. Ensemble Averaging

To mitigate the effect of statistical fluctuations of the lattice vibration field on the clustering of the electron dynamics, we perform the optimization independently on an ensemble of 10 wavefunction data sets, each generated using deformation potentials with different random initializations. The different clustering results are then consolidated into one phase diagram by taking the consensus of the ensemble, with the size of the dots in Figure 3 indicating the degree of consensus.

## Appendix B. Clustering in the Frozen Approximation

This appendix presents the clustering analysis results using the frozen field approximation, where the deformation potential is treated as static. This simplifies the system by ignoring the temporal evolution of lattice vibrations, allowing for a direct comparison with the dynamic scenario discussed in the main text.

Using the same *k*-means clustering technique as described in Appendix A, we simulate wavepacket evolution under static deformation potentials. These potentials are generated with identical parameters to those used for the dynamic scenario. We then analyze the resulting time-dependent wavefunctions using mean squared displacement (MSD) and inverse participation ratio (IPR) as features.

Figure A4 shows the phase diagram for LSCO derived from clustering in the frozen approximation. As in the dynamic case, three phases are identified. Supported by the arguments presented in the main text and especially Figure 6, Phases I and II are very similar to the dynamic case as the electron dynamics are highly adiabatic in these regimes. The key difference emerges in Phase III, the Anderson localization phase. Here, strong localization due to quantum interference in the static potential field leads to complete Anderson localization, contrasting sharply with the transient localization observed in the dynamic case.
